# Agreement of Cerebral State Index and Glasgow Coma Scale in Brain-Injured Patients

**DOI:** 10.5812/atr.15892

**Published:** 2014-03-30

**Authors:** Mehrdad Mahdian, Mohammad Reza Fazel, Esmaeil Fakharian, Hossein Akbari, Soroush Mahdian, Soheila Yadollahi

**Affiliations:** 1 Trauma Research Center, Kashan University of Medical Sciences, Kashan, IR Iran; 2 Student Research Committee, Arak University of Medical Sciences, Arak, IR Iran; 3 Shahid-Beheshti Hospital, Kashan University of Medical Sciences, Kashan, IR Iran

**Keywords:** Cerebrum, Glasgow Coma Scale, Brain Injury

## Abstract

**Background:**

Variables derived from electroencephalogram like cerebral state index (CSI) have been used to monitor the anesthesia depth during general anesthesia. Observed evidences show such variables have also been used as a detector of brain death or outcome predictor in traumatic brain-injured (TBI) patients.

**Objectives:**

The current study was designed to determine the correlation between Glasgow coma score (GCS) and CSI among TBI patients.

**Patients and Methods:**

In 60 brain-injured patients who did not need and receive sedatives, GCS and CSI were daily measured during the first ten days of their hospital stay. Correlation between GCS and CSI was studied using the Pearson's correlation test. The Gamma agreement coefficient was also calculated between the two variables for the first day of hospitalization.

**Results:**

A significant correlation coefficient of 0.611-0.796 was observed between CSI and GCS in a ten-day period of the study (P < 0.001). Gamma agreement coefficient was 0.79 (P < 0.001) for CSI and GCS for the first day of hospitalization. An increased daily correlation was observed in both CSI and GCS values. However, this increase was less significant in CSI compared with the GCS.

**Conclusions:**

A statistically significant correlation and agreement was found between GCS and CSI in the brain-injured patients and GCS was also found to be more consistent and reliable compared with CSI.

## 1. Background

Traumatic brain injury (TBI) is one of the major causes of post-injuries mortality and disability, worldwide (1). TBI is characterized by the loss of consciousness and Glasgow coma scale (GCS) is used for routine assessment of the patients' consciousness level. In non-traumatic unconsciousness like during anesthesia, variables derived from electroencephalogram (EEG) like bispectral index (BIS) and cerebral state index (CSI) have been widely used to monitor the depth of anesthesia objectively and quantitatively (2). Among these indices further studies have been undertaken based on BIS (3-5). Many investigators have tried to broaden the monitoring use of BIS for TBI patients (6, 7) and among those many studies found a significant correlation between BIS and GCS (8-11) and some did not (12). The CSI value which was introduced as a new instrument for measuring hypnotic depth in 2004, is passively derived from EEG and demonstrates a dimensionless score from 0 to 100 (13-16). Unlike BIS, few studies have been performed on CSI monitoring in brain-injured patients. This study was conducted to determine the correlation and agreement between GCS and CSI in unsedated traumatic brain- injured patients.

## 2. Objectives

The purpose of this study was to assess the degree of agreement and correlation between GCS as a subjective and CSI as an objective method to evaluate the consciousness in TBI patients.

## 3. Patients and Methods

This prospective observational study was performed after approval and informed consent in patients with mild (GCS 13-15), moderate (GCS 9-12) and severe (GCS 3-8) head injury. All patients had been admitted to the neurosurgical ICU of Shahid-Beheshti Hospital, Kashan University of Medical Sciences, Iran, between December 2011 and March 2012. Demographic characteristics, mechanism of head injury, GCS, computed tomography scan, and neurological examination at the time of admission were recorded. Patients demanding sedatives were excluded from the study. Since some of the patients underwent intracranial surgical operation, to eliminate the effect of anesthesia on consciousness indices, GCS and CSI were measured three hours after recovery from general anesthesia in these cases. Respiratory and metabolic disorders were considered as exclusion criteria, due to their potential effects on GCS, and patients with such problems did not enter the study. CSI was measured after the ICU admission and afterwards on a daily basis for the next nine days. CSI was measured using CSI monitor (CSI^™^Danmeter, Odese, Denmark). To measure CSI, the patient's skin was prepared by swapping with alcohol and wiping. Standard electrocardiogram electrodes (SKINTACT^®^, Leonard Long GmbH, Innsbruk, Austria) were then positioned as recommended by the manufacturer. After controlling of electrode impedance, the numerical value of CSI, ranging 0-100, was recorded for each patient by a nurse who was blinded to the study. Patients' neurological status was assessed using the GCS and recorded, at the moment of CSI measuring.

### 3.1. Statistical Analysis

Correlation between GCS and CSI was calculated using Pearson's correlation test. To eliminate the effect of surgical operation and patients' death on the study, adjustments regarding these factors were performed and partial correlation was calculated in addition to the crude correlation. Gamma agreement coefficient was also calculated between two variables for the first day of hospitalization. Mean and standard deviations for CSI and GCS values in brain injured patients in different days were also compared using t-test.

## 4. Results

**Figure 1.**
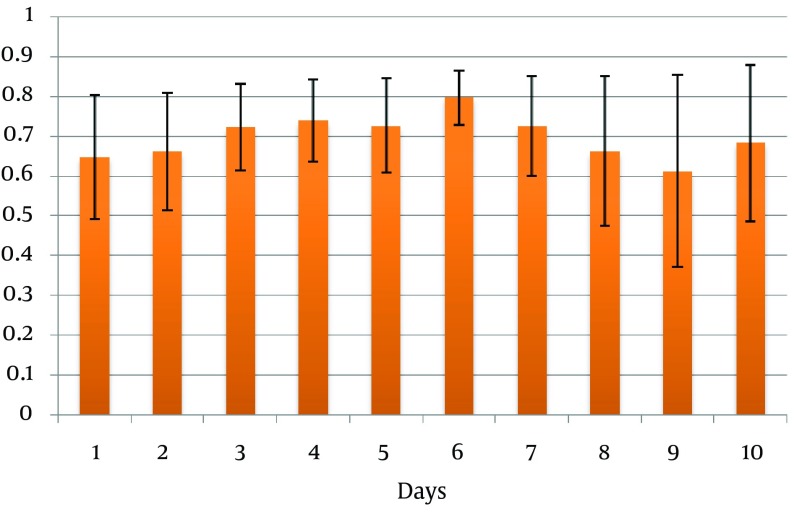
Correlation Coefficient and 95% Confidence Interval of GCS and CSI During Hospitalization

A total of 60 brain-injured patients (53 male and seven female cases) aged (mean ± SD) 33.4 ± 17.1 years were included in the study. The injury mechanism was road traffic accident in 49 cases, falling in nine and assault in two patients. Fourteen patients had mild injury, 13 had moderate and 33 had severe injuries. The majority of cases were admitted to the ICU due to subdural hematoma and during the course of the study 26 cases underwent surgical operation due to intracranial mass lesion and 17 patients (28.3%) expired due to their condition deterioration (Table 1). Ten sets of data were collected for each patient. An increased daily correlation was observed in both CSI and GCS values. However, this increase was less significant for CSI than that of the GCS. A significant correlation coefficient (crude correlation) of 0.611-0.796 between CSI and GCS was observed in the ten-day study. A significant partial correlation was also noted between the two indices regarding adjustment of surgical operation and death during the study period (Table 2). The highest correlation coefficient between GCS and CSI in all the patients was observed on the sixth day of hospitalization (r = 0.796, P < 0.001) (Figure 1). Gamma agreement coefficient for CSI and GCS was 0.79 (P < 0.001) for the first day of hospitalization. 

**Table 1.** Patients' Outcomes Regarding Their CT Scan-Based Diagnoses ^a^

Outcome Diagnosis	Death	Transfer to Post-ICU Ward	Sum
**Subdural hematoma**	8 (42.1)	11 (57.9)	19
**Epidural hematoma**	2 (11.8)	15 (88.2)	17
**Cerebral contusion**	2 (22.2)	7 (77.8)	9
**Diffuse axonal injury**	4 (40)	6 (60)	10
**Intracranial Hemorrhage**	1 (20)	4 (80)	5
**Sum**	17 (28.3)	43 (71.7)	60

^a^ All data are expressed as No (%).

**Table 2.** Correlation Between GCS and CSI in Different Days of Hospitalization (Including Crude and Adjusted Correlation Regarding Death and Surgical Operation)

Correlation, Days	Crude Correlation	Partial Correlation Adjusted for Death	Partial Correlation Adjusted for Surgical Operation
**1**	0.648	0.473	0.479
**2**	0.661	0.628	0.731
**3**	0.723	0.723	0.787
**4**	0.739	0.721	0.772
**5**	0.726	0.619	0.695
**6**	0.796	0.68	0.79
**7**	0.725	0.612	0.73
**8**	0.662	0.529	0.646
**9**	0.611	0.457	0.6
**10**	0.682	0.504	0.68

## 5. Discussion

The present study showed that there is a statistically significant correlation and agreement between GCS and CSI in patients with traumatic brain injury. Although we have found a correlation between GCS and CSI in patients during all days of hospitalization, interestingly, we found GCS was more consistent than the CSI.

Basically, cerebral state index has been developed for monitoring the depth of anesthesia during operations. Its predecessor, BIS, has been also used for monitoring of consciousness during the management of both traumatic and non-traumatic coma (6, 17). The CSI, like BIS, is a quantitative EEG derived measure index that provides a dimensionless score from 0 to 100 to characterize the level of hypnosis. Although EEG monitoring is the gold standard for intraoperative monitoring of cerebral ischemia but common monitoring of EEG is inconvenient and obtaining signals without artifacts may be difficult in the operating room and intensive care settings (6). Therefore, it is preferable to use simple tools to measure BIS or CSI. Unlike BIS that was used in many studies to evaluate the consciousness state and diagnosis of brain death in comatose patients (6, 7, 11, 18-21), limited clinical studies have been conducted on the CSI in this area. Of course there are series of studies showing values of CSI and BIS, which are not significantly different (22-24). However, as previously mentioned, BIS measurement has recently been used for patients with traumatic brain injuries with severity of the injury (6, 7, 10, 20). The findings of the present study are consistent with previous observations and suggested that CSI values also correlate with severity of brain injury. These findings also corroborate the ideas of Ming XU et al. (2011), suggested that CSI monitoring presumably is a reliable objective technique to predict consciousness level of patients following elective intracranial surgeries (25). However, the findings of the current study does not support the published study by Nasraway et al. (2002) (12) who compared BIS with the sedation-agitation scale (SAS) as a subjective tool for monitoring of consciousness in critically ill patients. They concluded that the correlation between SAS and BIS scores was less than optimum and unpredictable in a different group of ICU patients. This inconsistency with our results may be due to the nature of the two subjective tools. They used SAS while the index used in the present study was GCS. In addition, this study was performed on the brain-injured patients while they studied on a heterogeneous group of patients. Finally, a significant correlation and agreement was found between GCS and CSI in brain-injured patients. So, CSI can be used as an adjunct to GCS to evaluate the consciousness in these patients.
